# End-threaded intramedullary positive profile screw ended self-tapping pin (Admit pin) - A cost-effective novel implant for fixing canine long bone fractures

**DOI:** 10.14202/vetworld.2018.181-185

**Published:** 2018-02-13

**Authors:** Mitin Chanana, Adarsh Kumar, Som Prakash Tyagi, Amit Kumar Singla, Arvind Sharma, Uiase Bin Farooq

**Affiliations:** Department of Veterinary Surgery and Radiology, College of Veterinary and Animal Sciences, Chaudhary Sarwan Kumar Himachal Pradesh Krishi Vishvavidyalaya, Palampur – 176 061, Himachal Pradesh, India

**Keywords:** Admit pin, canine, end-threaded, fracture, intramedullary, orthopedics, pinning, positive profile

## Abstract

**Aim:**

The current study was undertaken to evaluate the clinical efficacy of end-threaded intramedullary pinning for management of various long bone fractures in canines.

**Materials and Methods:**

This study was conducted in two phases, managing 25 client-owned dogs presented with different fractures. The technique of application of end-threaded intramedullary pinning in long bone fractures was initially standardized in 6 clinical patients presented with long bone fractures. In this phase, end-threaded pins of different profiles, i.e., positive and negative, were used as the internal fixation technique. On the basis of results obtained from standardization phase, 19 client-owned dogs clinically presented with different fractures were implanted with end-threaded intramedullary positive profile screw ended self-tapping pin in the clinical application phase.

**Results:**

The patients, allocated randomly in two groups, when evaluated postoperatively revealed slight pin migration in Group-I (negative profile), which resulted in disruption of callus site causing delayed union in one case and large callus formation in other two cases whereas no pin migration was observed in Group-II (positive profile). Other observations in Group-I was reduced muscle girth and delayed healing time as compared to Group-II. In clinical application, phase 21^st^ and 42^nd^ day post-operative radiographic follow-up revealed no pin migration in any of the cases, and there was no bone shortening or fragment collapse in end-threaded intramedullary positive profile screw ended self-tapping pin.

**Conclusion:**

The end-threaded intramedullary positive profile screw ended self-tapping pin used for fixation of long bone fractures in canines can resist pin migration, pin breakage, and all loads acting on the bone, i.e., compression, tension, bending, rotation, and shearing to an extent with no post-operative complications.

## Introduction

Management of fractures through intramedullary fixation is regularly used in Veterinary Orthopaedics. The Steinmann pin appears to be the most often used material, either on its own or in combination, while the Rush pin, Kirschner wire, Kuntscher pin, and interlocking pin have all been employed depending on their indication. Unthreaded intramedullary pins alone cannot provide adequate traction and rotational stability, as they are weak against rotational and shearing forces [1]. Stack pin application partially prevents these disadvantages by opposing the horizontal crossing and bending forces [2], and it has been reported that combined plate-intramedullary pin application is successful in increasing axial and rotational ­stability [3]. Intramedullary interlocking nailing is used to achieve rigid repair which can counteract all forces and are entirely load bearing until callus formation [4]. Rotational stability can also be increased by cerclage wire, external fixation, interlocking pins, and trilam nails [5], or using a C-clamp on the plate [6,7] reported that stabilization of a Salter-Harris Type IV physeal fracture of the humeral condyle in a miniature pinscher was simplified by using Orthofix partially threaded Kirschner wire, with excellent clinical results. Partially threaded pins, having a negative profile ending create a weak point in the pin-thread junction, so if these pins are to be used, the junction must not be near the fracture line [8]. The pros and cons of various implants were taken into account and the innovation in terms of “end-threaded intramedullary positive profile screw ended self-tapping pin” (Admit pin) was conceived to minimize the complications of frequently used economic routine intramedullary pinning.

The aim of this study was to standardize the technique of application of end-threaded intramedullary pin for management of long bone fractures in canines and to evaluate the efficacy of end-threaded intramedullary pin in the management of long bone fractures in canines.

## Materials and Methods

### Ethical approvals

The research design is purely an applied clinical study, therefore the ethical approval from Institutional animal ethics committee was not mandatory. However, the broad outline of the work has been approved by the committee.

### Design and manufacturing of Admit pin

**Figure-1:**
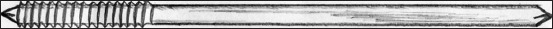
End-threaded intramedullary pin.

**Figure-2:**
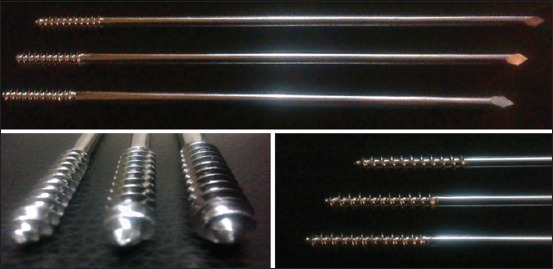
End-threaded intramedullary positive profile screw ended self-tapping pins of different sizes used in the study.

Intramedullary end-threaded pins (Figure-1) were manufactured from an iron based alloy-316L Stainless Steel, in an end-threaded fashion. Pins were produced in various diameters, ranging from 4.5 mm (major diameter)/4 mm (pitch diameter) to 7.5 mm/5.5 mm with a standard length of 9 inches. The distal end of the pin was designed with a positive profile self-tapping screw pointed end (Figure-2) to allow for ease of entry into the cancellous bone whereas the proximal end is kept trocared so that there is no need of a pilot hole in proximal fragment of bone when used either in retrograde or normograde fashion (Figure-2).

### Clinical application phase

In the present study, 25 client-owned dogs of different breeds, sex, and age presented in the Teaching Veterinary Clinical Complex of DGCN College of Veterinary and Animal Sciences, Palampur, with various long bone fractures were treated under two phases. The study was based on clinical cases, so there is no need of ethical approval as the cases were treated as per highest standard of the treatment at par with any national or international standards without harming or giving any unnecessary stress to the animals. All patients were subjected to clinical and radiological examinations preoperatively, those required mild sedation for pre-operative evaluation were administered acepromazine at 0.05 mg/kg B.Wt, I.M.; butorphanol tartrate at 0.2 mg/kg B.Wt, I.M. and atropine sulfate at 0.04 mg/kg B.Wt., S.C. The patient scheduled for surgery was given a standard protocol comprising injection butorphanol at 0.2 mg/kg B.Wt, I.V. and injection diazepam at 0.5 mg/kg B.Wt, I.V. followed by injection propofol (till effect, I.V.) and maintenance by isoflurane throughout the period of surgery. The diameter and length of the implant were determined by pre-operative radiographs, size and weight of the dog and intra-operative assessment. An open reduction with a lateral approach was the technique of choice for diaphyseal, epiphyseal, or metaphyseal femoral fracture repair; craniolateral approach was preferred for humeral fracture repair, and medial approach was preferred for tibial fracture repair.

### Research design

**Figure-3:**
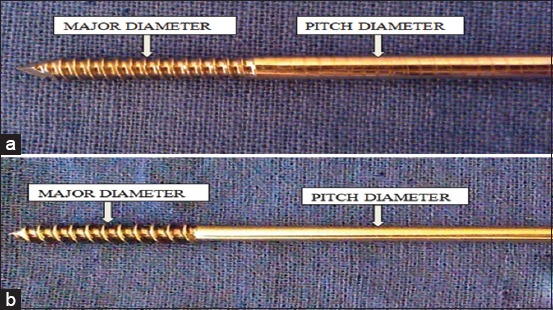
Positive and negative profile pins used for standardization. (a) Negative profile: Major diameter < Pitch diameter and (b) positive profile: Major diameter > Pitch diameter.

In the first phase, 6 dogs were used for technique standardization. These six client-owned dogs were randomly allocated into two groups. In Group-I, three dogs with long bone fracture (8, 3, and 1 years weighing 26 kg, 18 kg, and 20 kg, respectively) were implanted with end-threaded intramedullary negative profile pin (Figure-3) with a trocar end of 5.0/5.2 mm, Whereas in Group-II, three dogs with long bone fractures (18 months, 3 years, and 10 years weighing 16 kg, 20.5 kg, and 30 kg, respectively) were implanted with end-threaded intramedullary positive profile pin screw ended self-tapping pin of 4.5/6.5 mm (Figure-3).

On the basis of the results obtained from Phase-I, 19 client-owned dogs (age; 1.5 month - 9 years and body weight; 3.40-21 kg), clinically presented with fracture of different long bones (femur, humerus, and tibia) were implanted with end-threaded positive profile intramedullary pin with a screw end of size: 3.5/4.0 mm, 4.5/5.0 mm, and 4.5/6.5 mm.

## Results

In standardization phase, pins were removed after complete attainment of weight bearing by the animals with proper radiographic evidence of fracture healing in both groups. Evaluation at 21^st^ and 42^nd^ day postoperatively revealed slight pin migration in Group-I, which resulted in disruption of callus site causing delayed union in one case and large callus formation in other two cases whereas no pin migration was observed in Group-II. Other observations in Group-I was reduced muscle girth and delayed healing time as compared to Group-II. Rotational forces were not resisted by the implant used in Group-I. On the other hand, the implant used in Group-II successfully resisted rotational forces as the threads were embedded in distal cancellous bone firmly (Table-1). Partially threaded pins having a negative profile ending create a weak point in the pin-thread junction, so if these pins are to be used, the junction must not be near the fracture line. Positive profile pins do not have this problem because the threads are raised above the core diameter of the pin. Thus, there is no stress riser (weak point) at the thread non-thread interface and the implant appears very sturdy (Table-1).

**Table-1:** Comparison from available intramedullary implants.

Properties	Intramedullary implants		
	Steinmann pin	Available end-threaded pin	Pin designed for study
	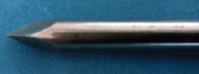	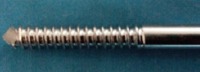	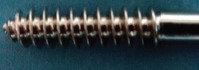
Threads	Absent	Negative profile, Fine thread	Selftapping, Positive profile, Coarse thread
Fitting into cancellous bone	Loosely fitted as threads are absent	Grip - better than Steinmann pin but threads were not of sufficient size to engage the cancellous bone	Grip would be better than other implants as threads are of sufficient size to engage the cancellous bone tightly
Pin thread junction	Absent, so no breakage of implant	Weak junction point, so pin breaks from the junction	Strong junction point, no breakage will be observed
Tip of pin	Trocar point	Trocar point	Screw point

In clinical application phase, 19 cases were studied (Table-2). The parameters evaluated, 21^st^ and 42^nd^ day postoperatively showed an initial decrease in muscle girth due to; removal of hematoma formed at the site, reduction of fractured bone fragments back to their normal position and post-operative resolution of inflammatory process. The gradual increase in muscle girth observed over a period of time was due to; increased blood supply to the site of fracture, regeneration of healthy muscle tissue and callus formation at the fracture site.

**Table-2:** Signalment of the animals during application phase.

Pin size (mm)	Cases	Age	Body weight (kg)
3.5/4.0	1	1 and ½ months	3.40
	2	2 months	3.70
	3	2 months	3.70
	4	3 months	4.70
	5	4 months	4.50
	6	4 months	4.30
	7	18 months	11.50
4.5/5.0	8	3 and ½ months	8.50
	9	4 months	9.75
	10	4 months	11.00
	11	6 months	7
	12	6 months	13
	13	6 months	13
	14	9 years	15
4.5/6.5	15	8 months	9.40
	16	11 months	21
	17	1 year	21
	18	4 months	7.5
	19	4 months	9.80

**Figure-4:**
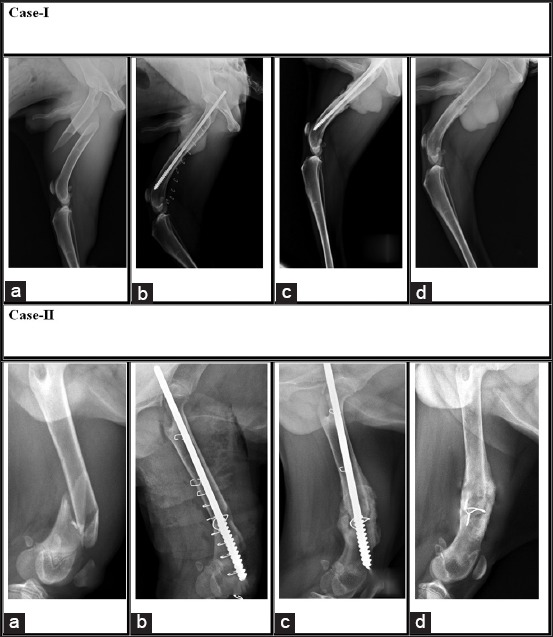
Serial radiographic observations in Group-II (positive profile pin) in two of the patients presented with a complete mid-diaphyseal simple spiral fracture of the right femur and closed complete comminuted distal diaphyseal fracture of left femur, respectively. Case-I: (a) pre-operative, (b) immediate post-operative, (c) 21 days post-operative, and (d) post pin removal. Case-II: (a) pre-operative, (b) post-operative, (c) 21 days post-operative, and (d) post pin removal.

In nearly all the cases, partial weight bearing was noticed on 10^th^-14^th^ post-operative day and nearly complete weight bearing on 21^st^-42^nd^ post-operative day with near-normal limb function. The radiographic follow-up revealed no pin migration in any of the cases, and there was no bone shortening or fragment collapse. When a bridge of periosteal callus was seen on 21^st^-30^th^ day postoperatively, it was classified as healed; all cases healed with a moderate degree of periosteal callus (Figure-4). There was no evidence of axial rotation or compression at either of the fracture lines in all the cases. Post-operative grading (42^nd^ day postoperatively) of functional limb usage on the basis of weight bearing and gait of animal showed that out of 19 cases, 12 showed normal limb usage, i.e., complete weight bearing with no sign of limping whereas 6 showed slight limping, i.e. good weight bearing with slight limping, but they also showed complete limb function within next 21 days. One animal died due to unknown cause before 42^nd^-day observation.

## Discussion

Bone as a biological material can absorb large amounts of load associated with the normal physiological activity, e.g., walking or running, whereas is less capable of tolerating a non-physiologic load [1,9]. Five individual forces acting on long bones are usually recognized, i.e., compression, tension, shearing, bending, and torsion [5,9]. These supraphysiologic forces exceed the ultimate strength of the bone, resulting in bone failure, often in a predictable fashion. The fracture configuration and degree of soft tissue trauma are due to the direction and magnitude of the force that is applied to the bone. The goal of any fracture treatment is early ambulation and complete return to full function [2,5]. In the present study, late weight-bearing was observed in Group-I implanted with negative profile pin where an early weight-bearing, i.e., within 21-42 days was observed in cases implanted with positive profile pins. The dogs appeared to bear weight on their limbs 5-15 days after the operation and functional recovery was seen to increase gradually and full weight-bearing without any signs of clinical complication was seen to occur after day 20. Delayed union (>60 days) was observed in Group-I with large callus formation in two patients, and moderate callus formation in one patient whereas normal union with gap healing was observed in Group-II with moderate callus formation. Gap healing (primary bone healing) occurs under rigid fixation in areas in which small gaps are present. In such healing, direct ossification takes place after in-growth of blood vessels, and original structure of bone is later restored by secondary Haversian remodeling in the long axis of the bone [10,11]. A large amount of external callus indicates the need for additional support beyond normal contours of the bone. This presents a delay in osseous healing over what can be obtained by stable fixation [12]. The amount of callus is in reverse relation to degree of stability at fracture site except in young growing animal [9]. The instability at the fracture site results in the large callus to prevent this motion [13]. Pin migration was observed in all the animals of Group-I whereas pin migration was not observed at any stage in the animals of Group-II. Rotational forces were not resisted by the implant used in Group-I. On the other hand, the implant used in Group-II successfully resisted rotational forces as the threads were embedded in distal cancellous bone firmly.

The fully-threaded Steinmann pins provide adequate rotational stability and prevention of pin migration when applied in normograde fashion in fractures of the femur, humerus, and tibia of cat [14]. The compression of the fractured segment at the fracture line was evident as the positive profile pin was screwed in distal fragment ensuring near normal continuity of the bone length and contours. Partially threaded pins having a negative profile ending create a weak point in the pin-thread junction, so if these pins are to be used, the junction must not be near the fracture line [8]. Positive profile pins do not have this problem because of the threads is raised above the core diameter of the pin. Thus, there is no stress riser (weak point) at the thread non-thread interface [15], and the implant appears very sturdy. In Group-I, to provide better stability to fractured fragments, the pin was snuggly fitted into medulla of the bone, covering maximum diameter thus obstructing medullary circulation to an extent which may be causing an increase in healing time. Fracture fixation alters the blood flow at the fracture site because the blood supply to the fracture hematoma, the bone cortex and the soft tissue is affected by the operative procedure used [16]. Whereas in Group-II, only threaded positive profile end of pin gets snuggly fitted into the spongy bone leaving the rest of medullary cavity (30-40%) unoccupied, thus allowing the medullary circulation to regenerate which may aiding in early healing [17].

## Conclusion

Hence, the end-threaded intramedullary positive profile screw ended self-tapping pin (Admit pin) used for fixation of long bone fractures in canines can resist pin migration, pin breakage and all loads acting on the bone. In addition, it can be easily used in field conditions in managing long bone fractures in canines, as compared to other orthopedic implants.

## Authors’ Contributions

AK conceived the innovation of design of the orthopedic implant and technical execution of the clinical research procedure. MC got the implant manufactured and executed the plan of research for his Masters dissertation. SPT used the implant in several clinical cases and contributed his critical experience. AKS gave anesthetic and surgical support during the procedures, AS helped in intraoperative fluoroscopy and radiographic follow-ups. UBF performed post-operative care. All authors read and approved the final manuscript.
